# Ginseng Total Saponins Reverse Corticosterone-Induced Changes in Depression-Like Behavior and Hippocampal Plasticity-Related Proteins by Interfering with GSK-3****β****-CREB Signaling Pathway

**DOI:** 10.1155/2014/506735

**Published:** 2014-01-09

**Authors:** Lin Chen, Jianguo Dai, Zhongli Wang, Huiyu Zhang, Yufang Huang, Yunan Zhao

**Affiliations:** ^1^Basic Medical College, Nanjing University of Chinese Medicine, Nanjing 210023, China; ^2^Laboratory of Pathological Sciences, Basic Medical College, Nanjing University of Chinese Medicine, Nanjing 210023, China; ^3^Key Laboratory of Brain Research, Basic Medical College, Nanjing University of Chinese Medicine, Nanjing 210023, China

## Abstract

This study aimed to explore the antidepressant mechanisms of ginseng total saponins (GTS) in the corticosterone-induced mouse depression model. In Experiment 1, GTS (50, 25, and 12.5 mg kg^−1^ d^−1^, intragastrically) were given for 3 weeks. In Experiment 2, the same doses of GTS were administrated after each corticosterone (20 mg kg^−1^ d^−1^, subcutaneously) injection for 22 days. In both experiments, mice underwent a forced swimming test and a tail suspension test on day 20 and day 21, respectively, and were sacrificed on day 22. Results of Experiment 1 revealed that GTS (50 and 25 mg kg^−1^ d^−1^) exhibited antidepressant activity and not statistically altered hippocampal protein levels of brain-derived neurotrophic factor (BDNF) and neurofilament light chain (NF-L). Results of Experiment 2 showed that GTS (50 and 25 mg kg^−1^ d^−1^) ameliorated depression-like behavior without normalizing hypercortisolism. The GTS treatments reversed the corticosterone-induced changes in mRNA levels of BDNF and NF-L, and protein levels of BDNF NF-L, phosphor-cAMP response element-binding protein (Ser133), and phosphor-glycogen synthase kinase-3**β** (Ser9) in the hippocampus. These findings imply that the effect of GTS on corticosterone-induced depression-like behavior may be mediated partly through interfering with hippocampal GSK-3**β**-CREB signaling pathway and reversing decrease of some plasticity-related proteins.

## 1. Introduction

Ginseng, the root of *Panax ginseng *C. A. Meyer (Araliaceae), is one of the most famous and valuable forms of traditional herbal medicine that has been widely applied for thousands of years. The early Chinese used ginseng as a general tonic and adaptogen to help the body to resist the adverse influence of a wide range of physical, chemical, and biological factors and to restore homeostasis [1]. Ginseng total saponins (GTS) are considered the principal bioactive ingredients behind claims of ginseng efficacy [2]. Recently, ginseng and ginsenosides have been shown to have several beneficial functions in the brain, including antidepressant or antistress effects. Our previous studies have shown that the water-based extract of ginseng exhibited protection against the hypercortisolism-induced impairment of hippocampal neurons without reversing the increased plasma corticosterone level [3, 4]. Some researchers reported that acute ginsenoside Rg1 treatment had antidepressant activity, as shown in a forced swimming test (FST) and a tail suspension test (TST) [5]. The antidepressant effects of ginsenosides administrated subacutely to normal mice or chronically to the chronic-mild-stress (CMS-) treated rats were also demonstrated in other studies [6, 7]. A study on immobilization-stressed gerbils has indicated the antistress effects of GTS and the ginsenosides Rg3 and Rb1 [8]. However, negative antidepressant and antianxiety results of ginseng were also reported [9].

The leading hypothesis on depression suggests that structural plasticity and neurotrophic factors are critical for mediating behavioral responses to antidepressants. Neurofilament light chain (NF-L) is a reliable marker of structural plasticity that indicates the impairment of neurons at the molecular level. NF-L is a subunit of neurofilaments (NFs). These NFs are neuron-specific cytoskeletal filaments found in most mature neurons. NFs provide structural support for neurons and their synapses as well as maintaining and regulating neuronal cytoskeletal plasticity by regulating neurite outgrowth, axonal caliber, and axonal transport [10]. Brain-derived neurotrophic factor (BDNF) is a key regulator of neuronal plasticity. It has been reported to strongly influence synaptogenesis, spine formation [11], neuronal survival [12], 1ong-term potentiation, neuronal excitability [13], and adult hippocampal neurogenesis [14]. The transcription of several genes such as BDNF is directed by activating the phosphorylation of the cAMP response element-binding protein (CREB) (Ser133) [15]. CREB is regarded as a key nucleoprotein related to depression and antidepressant treatments [16]. A growing number of studies have demonstrated that ginseng or ginsenosides can effectively upregulate the expression of these plasticity-related proteins. It is reported that the antidepressant activity of GTS in the CMS-treated rats may be partially mediated by enhancing BDNF expression in the hippocampus [6]. Chronic ginsenoside treatment could upregulate the expression of hippocampal plasticity-related proteins, including BDNF and phospho-CREB (Ser133) in aged mice [17, 18]. Our previous work on the water-based extracts of ginseng demonstrated the neuroprotective action of ginseng on hypercortisolism-induced hippocampal impairment by reversion of NF-L, BDNF, and some other plasticity-related proteins [3, 4].

The upstream signaling molecules and transduction pathways of CREB-BDNF are complex; among these, glycogen synthase kinase-3 (GSK-3) is a newly reported inhibitory signaling molecule [19–21]. This kinase was originally identified as a key enzyme of glucose metabolism. GSK-3 is a recognized broadly influential enzyme that affects a diverse range of biological functions because it regulates a large group of transcription factors and transcriptional modulators [22]. The discovery of the direct inhibition of GSK-3 by the mood stabilizer lithium [23] suggested that GSK-3 may be associated with the pathophysiology of mood disorders. Some GSK3 inhibitors have been reported to produce antidepressant-like effects in preclinical animal models [24–26]. GSK-3 exists in two closely related isoforms, namely, GSK-3*α* and GSK-3*β*. The constitutively active GSK-3*β* is an important regulatory protein involved in many intracellular signaling pathways related to neuroplasticity [27]; however, its activity is inhibited by Ser9 phosphorylation. Previous studies have shown that hippocampal GSK-3*β* activity significantly increased in CMS-treated rats or patients with major depressive disorders [28, 29], and antidepressant behavior was observed in CMS rats treated with GSK-3*β* inhibitors [28]. The overexpression of GSK-3*β* in the hippocampal dentate gyrus of CMS-treated mice caused prodepressant-like behavior [30]. Nevertheless, whether mice lacking one copy of the gene encoding GSK-3*β* exhibit less immobility time in the FST than the wild-type littermates is controversial [31, 32]. Although several animal studies on Alzheimer's disease have revealed that the ginsenoside Rb1 increases brain GSK-3*β* activity in vivo [33] and in vitro [34], fewer reports have investigated such effects in depression model animals. In the present study, we firstly observed the effects of chronic GTS treatment on depression-like behavior and some hippocampal plasticity-related proteins in male C57BL/6N mice, then investigated the aforementioned effects in the corticosterone-induced mouse depression model, and explored the underlying mechanism with respect to the GSK-3*β*-CREB signaling pathway.

## 2. Materials and Methods

### 2.1. Preparation and Quality Assessment of GTS

Chinese ginseng, specifically the root of *Panax ginseng*, was purchased from Beijing Tong Ren Tang Group Co., Ltd. (Beijing, China). The air-dried ginseng (150 g) was powdered and decocted thrice with 1.2 L of deionized water (for 1 h during each decoction). The resulting liquid was filtered and concentrated. The concentrated sample was then diluted with deionized water to a relative density of 1.06 g mL^−1^ and stored at 4°C until macroporous adsorption resin separation.

Following the methodology provided by China Pharmacopoeia 2010 [35], the ginseng water decoction was pumped through a fixed-bed column (20 mm × 300 mm) filled with 50 g of D101 macroporous adsorption resin (dry weight) at 50 mL h^−1^. When the adsorption reached equilibrium, 10 bed volumes of distilled water were pumped through the column to remove the contaminants at a rate of 250 mL h^−1^. Subsequently, 5 bed volumes of 60% aqueous ethanol were used to elute the ginsenosides in isocratic mode at a flow rate of 60 mL h^−1^. The eluent was collected and dried under vacuum at 60°C to produce the purified GTS (3.716 g).

For quality control, ultrahigh-performance liquid chromatography with a charged aerosol detector was applied to quantify the marker components. The total saponin content was estimated using the colorimetric method with a vanillin-vitriol system [36]. As shown in Table 1, the total saponin content of GTS was 66.9% ± 1.5%. The total amount of all representative ginsenosides Re, Rd, and Rg1 was approximately 10%.

### 2.2. Animals

Male C57BL/6N mice (weighing 18 g to 20 g, from the Laboratory Animal Center of Nanjing Medical University, Nanjing, China) were allowed 1 week to adapt to the laboratory environment before the actual experiments. Groups of 4–6 animals were housed in each cage with a 12 h light/12 h dark cycle (lights on between 7:00 and 19:00), at a constant room temperature of 22 ± 1°C, with free access to food and tap water. The animals were treated according to the Guidelines of Accommodation and Care for Animals formulated by the Chinese Convention for the Protection of Vertebrate Animals Used for Experimental and Other Scientific Purposes. Every effort was made to minimize the suffering and the number of animals used for the experiment.

### 2.3. Experimental Designs (Figure 1)

#### 2.3.1. Experiment 1

A total of 40 mice were divided into 5 matched groups (*n* = 8 per group), that is, the control group, 10 mg kg^−1^ d^−1^ fluoxetine (FLU) group, 50 mg kg^−1^ d^−1^ GTS (GTSH) group, 25 mg kg^−1^ d^−1^ GTS (GTSM) group, and 12.5 mg kg^−1^ d^−1^ GTS (GTSL) group. The GTS or fluoxetine (purchased from Venturepharm Co., Ltd. (China; purity, 99%)) was dissolved in distilled water and administrated by gavage to the respective group in a volume of 10 mL kg^−1^ once daily during 8:00 a.m.–12:00 a.m., for 3 weeks. The control group received distilled water. The administered doses were selected based on previous reports that GTS or fluoxetine could effectively produce physiological and behavioral effects on rodents. On day 20 and day 21, the depression-like behavior of the mice was observed via a FST and a TST at 4:00 p.m.–8:00 p.m., respectively. Twenty-four hours after the TST, all mice were quickly sacrificed to obtain hippocampal tissue samples for Western blot analysis.

#### 2.3.2. Experiment 2

Another total of 72 male C57BL/6N mice were used and divided into 6 matched groups (*n* = 12 per group): (1) the control group, (2) the corticosterone (CORT) group, (3) the corticosterone + 10 mg kg^−1^ d^−1^ fluoxetine (CORT + FLU) group, (4) the corticosterone + 50 mg kg^−1^ d^−1^ GTS (CORT + GTSH) group, (5) the corticosterone + 25 mg kg^−1^ d^−1^ GTS (CORT + GTSM) group, and (6) the corticosterone + 12.5 mg kg^−1^ d^−1^ GTS (CORT + GTSL) group. Corticosterone (Sigma) was suspended in physiological saline containing 0.1% dimethyl sulfoxide and 0.1% Tween-80, and this suspension was administered subcutaneously (20 mg kg^−1^) once daily at random times during 8:00 a.m.–12:00 a.m. for 22 days to induce the depression model. The control group received subcutaneous administration of the vehicle. After each corticosterone injection, GTS or fluoxetine was administered once daily to the respective group by gastric gavage, whereas the control and CORT groups orally received distilled water. On day 20 and day 21, the FST and the TST were performed during 4:00 p.m.–8:00 p.m., respectively. At 6 h after the last dose of corticosterone (day 22), all mice were quickly sacrificed to obtain venous blood samples and bilateral hippocampal tissue samples. Blood plasma was isolated by centrifugation at 3000 rpm and stored at −20°C until the corticosterone concentrations were assayed. Six samples of bilateral hippocampal tissue selected randomly from each group were prepared for Western blot analysis and the others for real-time PCR analysis.

### 2.4. Behavioral Tests

Behavioral tests were performed in JLBehv-FSG-4 sound insulation boxes with the DigBehav animal behavior video analysis system (Shanghai Jiliang Software Technology Co. Ltd., Shanghai, China). DigBehav can automatically record and analyze animal movements to provide total immobility times during the FST and TST. Depression-like behavior was inferred from the increasing time spent immobile during these tests. The FST method was similar to that described by Porsolt et al. [37] with a slight modification. The mice were placed individually in 10 cm deep water at ambient temperature (25 ± 1°C) in a 2000 mL glass beakers and were allowed to swim for 5 min. The duration of immobility was recorded during the last 4 min of the test. The TST method was similar to that described by Steru et al. [38]. After the FST, the mice were allowed to rest for 24 h. Each mouse was then suspended on the edge of a shelf at 58 cm above the bottom of the sound insulation box, using adhesive tape placed approximately 1 cm from the tip of the tail. The animals were allowed to hang for 6 min, and the duration of immobility was recorded during the last 4 min of the test.

### 2.5. Corticosterone Assays

Serum corticosterone was assayed using the AssayMax Corticosterone ELISA kit (Assaypro, Catalog no. EC3001-1; http://www.assaypro.com/), according to the instructions of the manufacturer. This kit employs a quantitative competitive enzyme immunoassay technique. A standard and/or serum sample (25 *μ*L) was added to each well of a 96-well microplate precoated with a corticosterone-specific polyclonal antibody, followed by the addition of 25 *μ*L of biotinylated corticosterone. After 2 h of incubation, the wells were washed five times with the wash buffer. Streptavidin-peroxidase (50 *μ*L) was then added to each well, and the mixtures were incubated for 30 min. After washing five times with wash buffer, 50 *μ*L of the chromogen substrate was added per well. The reaction mixtures were incubated until the optimal blue color density was observed. After adding 50 *μ*L of the stop solution, the absorbance was read immediately on a microplate reader at a wavelength of 450 nm. Finally, the mean value of the triplicate readings for each standard and serum sample was calculated, and the unknown sample concentration was determined from the standard curve.

### 2.6. Real-Time PCR Analysis

Total RNA from bilateral hippocampal tissue was extracted using Trizol reagent (Invitrogen). cDNA was synthesized with 2 *μ*g of total RNA using the RevertAid Transcript First-Strand cDNA Synthesis Kit (Fermentas, K1622). Quantitative real-time PCR was performed using the SYBR Green Master Mix (Fermentas, K0222) in the StepOne TM Real-Time PCR System (ABI, American). The sequences of primers were BDNF forward: 5′-GGTCACAGCGGCAGATAAAAAGAC-3′, reverse: 5′-TTGGGTAGTTCGGCATTGCGAG-3′; NF-L forward: 5′-GTTCAAGAGCCGCTTCACCG-3′, reverse: 5′-CCAGGGTCTTAGCCTTGAGCAG-3′; GAPDH forward: 5′-TGAAGGTCGGAGTCAACGGATTTGGT-3′, reverse: 5′-CATGTGGGCCATGAGGTCCACCAC-3′. The following thermal cycling conditions were used: initial denaturation at 95°C for 10 min, followed by 40 cycles of denaturation at 95°C for 15 s then annealing and extension, both at 60°C for 1 min. The amplification of only a single sequence was verified by the dissociation curve of each reaction. All experiments were performed in triplicate, and the average threshold cycle (Ct) value was the extreme Ct value of the sample. The mRNA expression of BDNF or NF-L was calculated relative to the housekeeping gene GAPDH using the 2^−ΔCt^ method [ΔCt = Ct_(the  target  gene)_ − Ct_(GAPDH)_].

### 2.7. Western Blot Analysis

Bilateral hippocampal tissue samples were homogenized at 4°C in 0.5 mL of lysis buffer containing 50 mM Tris-HCl, 0.1% sodium dodecyl sulfate (SDS), 1% nonidet-P40 (NP-40, Sigma), 1 mM EDTA, 150 mM NaCl, 1 mM phenylmethylsulfonyl fluoride (Sigma), 1 mM NaF, 1 mM Na_3_VO_4_, 1 *μ*g mL^−1^ aprotinin (Sigma), and 1 *μ*g mL^−1^ leupeptin (Sigma) (pH 7.5). Aliquots of the clarified homogenized liquid, containing 75 *μ*g of protein, were denatured at 95°C for 5 min in a sample buffer containing 1% SDS, 1% dithiothreitol (Sigma), 10 mM Tris-HCl, 10% glycerol, and 1 mM EDTA (pH 8.0). The sample proteins were then separated by 12% SDS-polyacrylamide gel electrophoresis and transferred to polyvinylidene fluoride membranes (Bio-Rad). The primary antibodies used to examine the changes in protein expression included the rabbit polyclonal anti-BDNF antibody (1 : 200, Abcam, ab6201), the mouse monoclonal anti-NF-L antibody (1 : 500, Invitrogen, 13-0400), the rabbit monoclonal anti-CREB (1 : 1000, Cell signal, 9197S), the rabbit monoclonal phospho-CREB (Ser133) (1 : 1000, Cell signal, 9198S), the rabbit monoclonal anti-GSK-3*β* (1 : 1000, Cell signal, 9315S), the rabbit monoclonal anti-phospho-GSK-3*β* (Ser9) (1 : 1000, Cell signal, 9323S), and the mouse monoclonal anti-*β*-actin (1 : 2000, Sigma, A1978). The secondary antibodies included the horseradish peroxidase-conjugated goat anti-mouse IgG (1 : 4000, GeneScript) and the goat anti-rabbit IgG (1 : 4000, GeneScript). Immunoblotting was detected by enhanced chemiluminescence (Amersham) and analyzed using an FR-200A Electrophoresis Image Analysis System (Furi, Shanghai, China). The values of the BDNF, NF-L, CREB, and GSK-3*β* levels were normalized against the amount of *β*-actin obtained from the same sample. The phospho-GSK-3*β*/GSK-3*β* and phospho-CREB/CREB were calculated to reflect the activity of GSK-3*β* and CREB. Three protein samples per animal were examined for each target protein.

### 2.8. Statistical Analysis

Data were expressed as the mean ± SEM for the indicated number of experiments and analyzed using the Statistical Package for Social Sciences computer program (version 13.0). The statistical significance of the results was determined using one-way ANOVA, followed by Tukey's post hoc tests. The significance level was set at *P* ≤ 0.05 for all statistical comparisons.

## 3. Results

### 3.1. Experiment 1: Effects of Chronic GTS Treatments on Depression-Like Behavior and Hippocampal Protein Levels of BDNF and NF-L in Male C57BL/6N Mice

**Figure 2:**
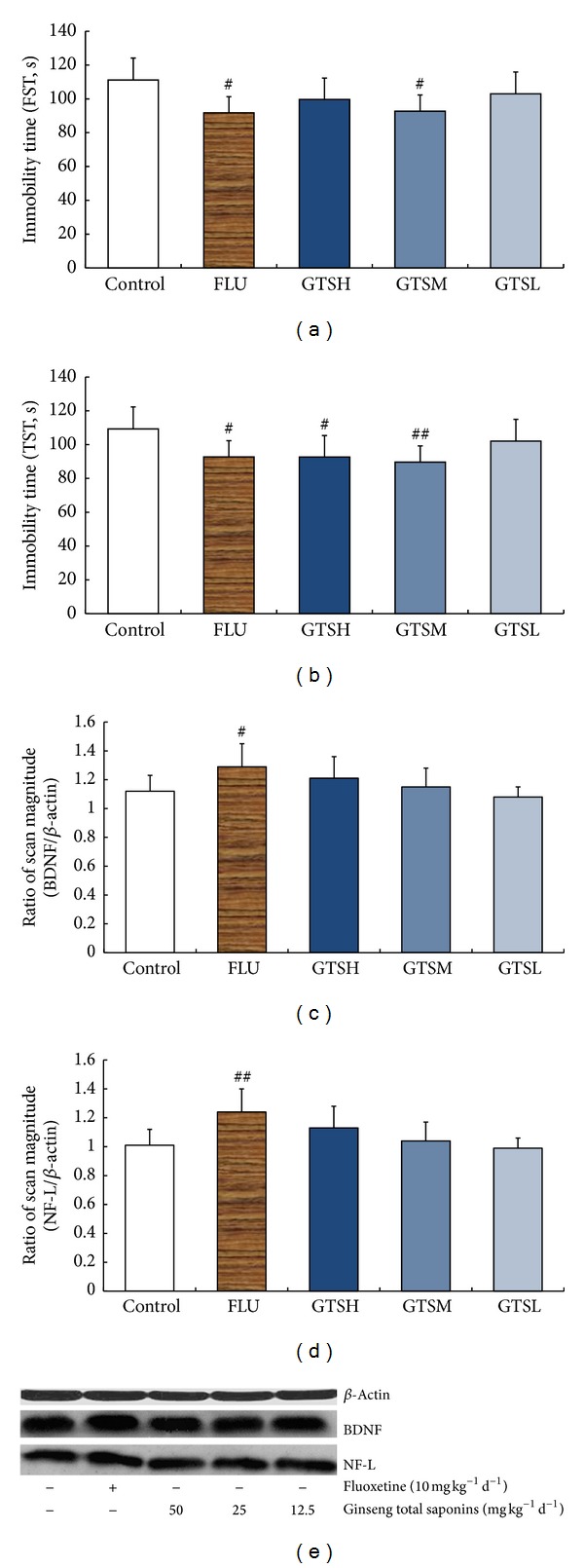
Effects of fluoxetine or different doses of ginseng total saponins on the depression-like behavior in the FST (a) and TST (b) and hippocampal protein levels of BDNF (c, e) and NF-L (d, e). ^##^ *P* < 0.01 versus the control group; ^#^ *P* < 0.05 versus the control group.

Figure 2 summarizes the results of chronic GTS treatments on male C57BL/6N mice. Results of behavioral tests showed significant immobility time differences among the groups in both FST (*F*_4,35_ = 3.718, *P* < 0.05) and TST (*F*_4,35_ = 4.341, *P* < 0.01). Chronic FLU or GTSM treatment significantly reduced immobility time in FST (*P* < 0.05 versus control). In the TST, the groups of FLU, GTSH, and GTSM all had markedly decreased immobility time (*P* < 0.05, *P* < 0.05, and *P* < 0.01, resp., versus control). The one-way ANOVA test revealed a main effect of groups for BDNF (*F*_4,35_ = 4.194, *P* < 0.01) and NF-L (*F*_4,35_ = 5.662, *P* < 0.01) protein expression in hippocampus. Multiple comparison tests revealed that BDNF and NF-L protein levels in the hippocampus were not altered statistically after chronic GTS treatments but were significantly increased in the FLU group (*P* < 0.05 and *P* < 0.01, resp., versus control).

### 3.2. Experiment 2: Effects of Chronic GTS Treatments in the Corticosterone-Induced Mouse Depression Model

#### 3.2.1. Higher or Moderate Dose of GTS Reversed the Increased Depression-Like Behavior Induced by Corticosterone

**Figure 3:**
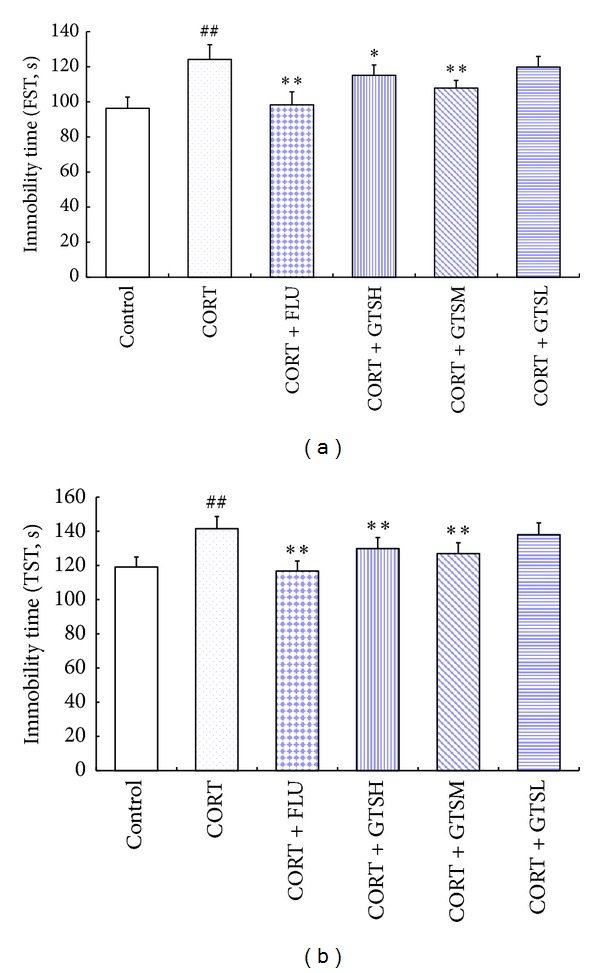
Effects of fluoxetine or different doses of ginseng total saponins on the depression-like behavior in the FST (a) and TST (b) in the corticosterone-induced mouse depression model. **P < 0.01 versus the CORT group, *P < 0.05 versus the CORT group, and ^##^ *P* < 0.01 versus the control group.

The immobility time differed significantly among the groups in both FST (*F*_5,66_ = 36.802, *P* < 0.01) and TST (*F*_5,66_ = 22.430, *P* < 0.01; Figure 3). The post hoc test revealed that the corticosterone injections induced a significant increase in the immobility time during FST and TST, as compared with the control group (*P* < 0.01). The aforementioned immobility time was almost reversed by simultaneous treatment with FLU, GTSH, or GTSM.

#### 3.2.2. All Doses of GTS Had No Significant Effects on Normalizing Hypercortisolism

**Figure 4:**
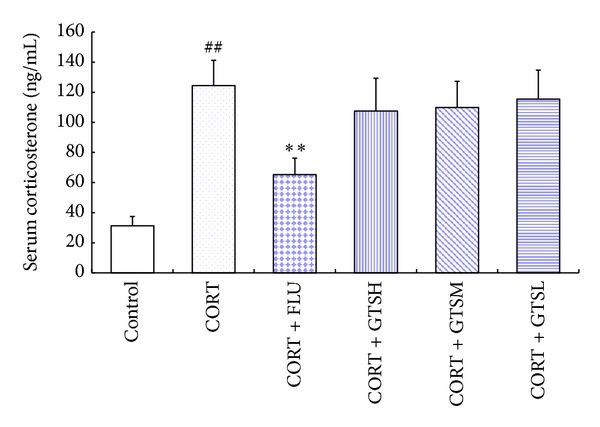
Effects of fluoxetine or different doses of ginseng total saponins on serum corticosterone in the corticosterone-induced mouse depression model. **P < 0.01 versus the CORT group; ^##^ *P* < 0.01 versus the control group.

The one-way ANOVA test revealed a significant effect of the groups on serum corticosterone levels (*F*_5,66_ = 60.492, *P* < 0.01). Multiple comparison tests revealed that the serum corticosterone level increased approximately fourfold in the CORT group, as compared with the control group (*P* < 0.01, Figure 4). Compared with the CORT group, the FLU group had significantly decreased serum corticosterone level (*P* < 0.01). By contrast, all three doses of GTS had no significant effect (*P* < 0.05).

#### 3.2.3. Certain Doses of GTS Ameliorated the Hippocampal mRNA Levels of BDNF and NF-L in the Corticosterone-Induced Mouse Depression Model

**Figure 5:**
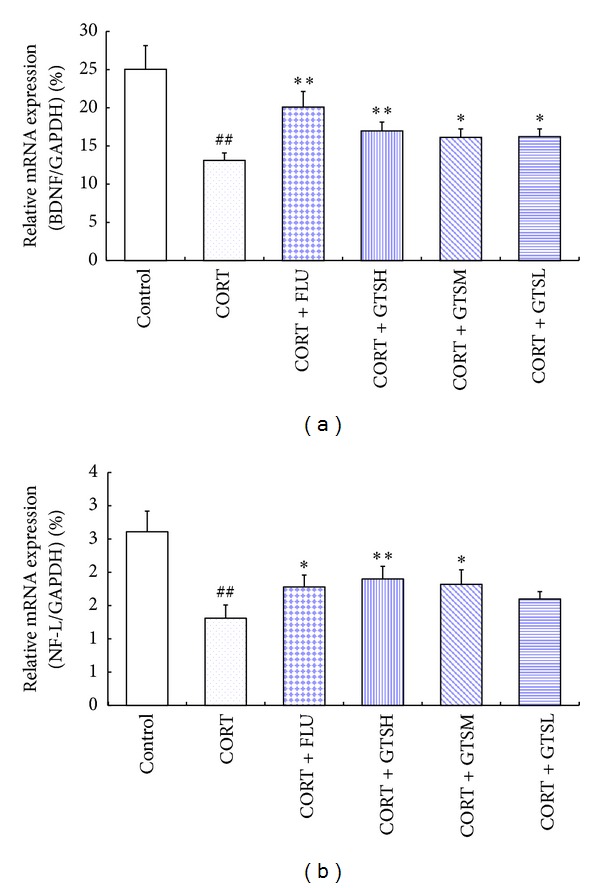
Effects of fluoxetine or different doses of ginseng total saponins on the hippocampal mRNA levels of BDNF (a) and NF-L (b) in the corticosterone-induced mouse depression model. **P < 0.01 versus the CORT group, *P < 0.05 versus the CORT group, and ^##^ *P* < 0.01 versus the control group.

The relative target gene mRNA levels of the groups are shown in Figure 5. The ANOVA tests showed a significant effect of the groups in the hippocampal mRNA level of BDNF or NF-L (*F*_5,30_ = 45.659 and *F*_5,30_ = 22.826, resp., *P* < 0.01). Post hoc comparisons revealed that the corticosterone injections significantly decreased the hippocampal mRNA levels of BDNF and NF-L compared with the control group (*P* < 0.01). Compared with the CORT group, FLU, GTSH, and GTSM significantly upregulated mRNA levels of BDNF (*P* < 0.01, *P* < 0.01, and *P* < 0.05, resp.) and NF-L (*P* < 0.05, *P* < 0.01, and *P* < 0.05, resp.). GTSL also reversed corticosterone-induced decrease of BDNF mRNA (*P* < 0.05 versus CORT).

#### 3.2.4. Certain Doses of GTS Promoted the Hippocampal Protein Levels of GSK-3*β* Inhibitory Phosphorylation, CREB Activation, BDNF, and NF-L in the Corticosterone-Induced Mouse Depression Model

**Figure 6:**
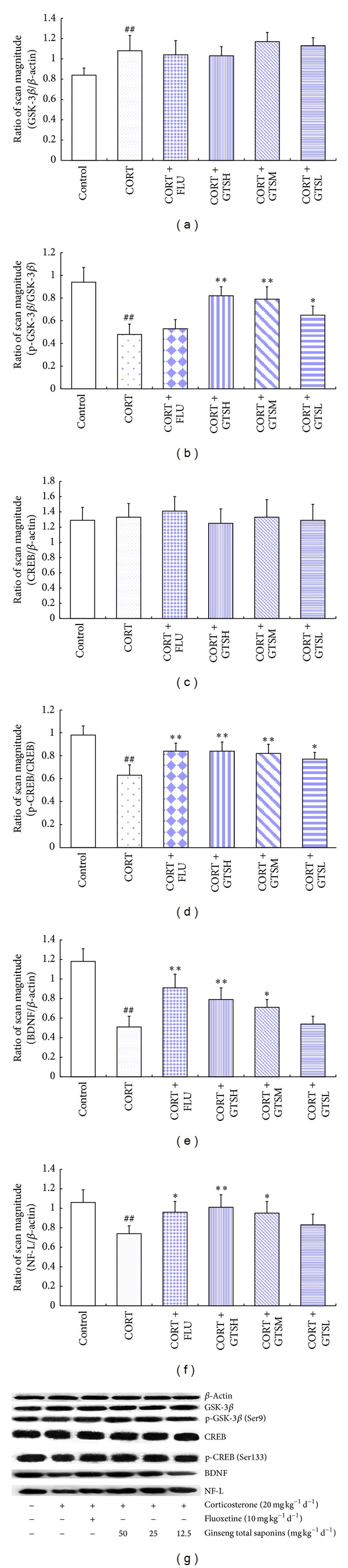
Hippocampal GSK-3*β*, p-GSK-3*β*, CREB, p-CREB, BDNF, and NF-L protein levels in the corticosterone-induced mouse depression model were determined by Western blot analysis. The values of GSK-3*β* (a), CREB (c), BDNF (e), and NF-L (f) levels were normalized against the amount of *β*-actin, while the values of p-GSK-3*β* (Ser9) (b) and p-CREB (Ser133) (d) were normalized against the amount of GSK-3*β* and CREB, respectively. **P < 0.01 versus the CORT group, *P < 0.05 versus the CORT group, and ^##^ *P* < 0.01 versus the control group.

As shown in Figure 6, the hippocampal protein level of GSK-3b was significantly higher in the CORT group compared with the control group (*P* < 0.01). Compared with the CORT group, neither of all three doses of GTS nor FLU influenced hippocampal GSK-3*β* expression. The ratio of phospho-GSK-3*β* (Ser9) and GSK-3*β* was statistically different among groups (*F*_5,30_ = 21.447, *P* < 0.01). The ratio drastically declined in the CORT group compared with the control (*P* < 0.01), GTSH (*P* < 0.01), GTSM (*P* < 0.01), and GTSL (*P* < 0.05) groups. However, FLU did not enhance this ratio, as compared with the CORT group.

No statistical difference was observed among the groups in the expression of hippocampal CREB protein (*F*_5,30_ = 0.455, *P* = 0.806), but the ratio of phospho-CREB (Ser133) and CREB among the groups was statistically significant (*F*_5,30_ = 13.556, *P* < 0.01). Post hoc comparisons showed that the ratio of phospho-CREB (Ser133) and CREB was lower in the CORT group than that in the control group (*P* < 0.01), and FLU, GTSH, GTSM, or GTSL treatment significantly increased the ratio (*P* < 0.01, *P* < 0.01, *P* < 0.01, and *P* < 0.05, resp., versus CORT).

The hippocampal protein levels of BDNF or NF-L in the CORT group were statistically lower than that in the control group (*P* < 0.01), whereas daily treatment with GTSH (*P* < 0.01, BDNF; *P* < 0.01, NF-L), GTSM (*P* < 0.05, BDNF; *P* < 0.05, NF-L), or FLU (*P* < 0.01, BDNF; *P* < 0.05, NF-L) during chronic corticosterone injections partially reversed the aforementioned detrimental effects.

## 4. Discussion

### 4.1. Repeated Corticosterone Injections Induced Depression Model

Among the existing animal models of depression, the CMS model is the most widely used one. This model is utilized in a wide range of stressful stimuli to activate the hypothalamic-pituitary-adrenal axis that simulates the presumed etiology of the disorder. CMS has been demonstrated to be valid and reliable; however, the procedure is time consuming, laborious, and sensitive to surroundings, which consequently increases experimental variability [39]. The corticosterone-induced depression model is built on the hypothesis that high levels of glucocorticoid contribute to the etiology of depressive symptomatology. Several studies have indicated that repeated corticosterone injections elicit an increase in immobility behavior during FST and/or TST in rodents [40–42]. Furthermore, the depression-like behavior induced by repeated corticosterone injections has been demonstrated to be independent of the changes in locomotor activity or muscle strength [43]. Chronic administration of corticosterone in rodents not only results in anhedonic- and helplessness-like behaviors that are persistent yet reversible by chronic antidepressant treatment, but also influences molecular targets hypothesized to contribute to depression [44]. Repeated corticosterone injections in male C57BL/6N mice have been reported to mimic the behavioral and neurochemical changes associated with depression and regarded as a convenient and reliable depression model [42]. Although this model is not very similar to the CMS model, it is thought to be an alternative method to the CMS model [44].

### 4.2. Effects of GTS on Depression-Like Behavior and Hippocampal Plasticity-Related Proteins

Previous studies have revealed that ginsenosides administrated acutely or subacutely to normal mice exhibited antidepressant-like effects [5, 6]; the present study (Experiment 1) showed that chronic GTS treatment (50 and 25 mg kg^−1^ d^−1^) to normal mice significantly reduced the immobility time in FST and/or TST. Since ginseng and ginsenosides were reported to have no effect on increasing the spontaneous locomotor activity in normal mice [5, 6, 9], the reduced immobility time may account for the antidepressant activity. Interestingly, the present results seem to stand opposite to another research which showed that chronic treatment with ginseng extract (500 mg kg^−1^ d^−1^) did not have an effect on immobility time in FST [9]. This contrast may be ascribed to the differences in many experimental parameters, such as component, dose, and delivery of treatment.

The behavioral results from the present study (Experiment 2) are in agreement with previous work examining the effects of chronic ginsenosides treatment in CMS-treated rodents [5–7]. To the best of our knowledge, the present study is the first to testify the antidepressant effects of GTS in the corticosterone-induced mouse depression model. The results (Experiment 2) confirm the previous reports that chronic corticosterone injections induce depression-like behavior and hippocampal impairment in mice, considering the increased immobility time in behavioral tests and the reduced expression of BDNF and NF-L in the hippocampus. Moreover, these adverse effects of corticosterone can at least be partially removed by GTS treatment (50 and 25 mg kg^−1^ d^−1^), whereas lower dose of GTS (12.5 mg kg^−1^ d^−1^) did not have significant ameliorating effects on BDNF and NF-L protein levels as well as on the behavior tests. Since chronic GTS treatment did not significantly increase BDNF or NF-L expression in the hippocampus of normal mice, the protective role of GTS against corticosterone-induced depression-like behavior may result from reversing corticosterone-induced decrease in these plasticity-related proteins, thereby implying the recovery of neuroplasticity.

### 4.3. Effect of GTS on Serum Corticosterone Levels in the Corticosterone-Induced Mouse Depression Model

Results from the serum corticosterone assays showed that the repeated corticosterone administration induced hypercortisolism. In addition, fluoxetine, a classical antidepressant applied in this experiment as a positive control, could effectively depress the increased level of serum corticosterone. However, all three doses of GTS, whether or not they exhibited protective role in depression-like behavior and neuroplasticity, had no such effect. These results are similar to our previous study on water-based ginseng extracts [3, 4] but are inconsistent with other studies on ginsenoside that employed the CMS depression model [5, 7]. This discrepancy may be caused by several parameters in the experimental design, including the different depression models, types of saponins, and dosage. In the corticosterone-induced mouse model, GTS did not normalize the high level of serum corticosterone although it showed antidepressant-like effects. Therefore, its preventive mechanism may involve modulating the central nervous system (CNS) targets instead of the peripheral antiglucocorticoids.

### 4.4. Effect of GTS on the Signaling Pathway of GSK-3*β*-CREB-BDNF in the Corticosterone-Induced Mouse Depression Model

A growing body of evidence suggests that neuroplasticity-related signaling pathways may be involved in the pathophysiology of depression and in the mechanisms of antidepressant action [45]. The present study addresses this issue by investigating the involvement of the CREB-BDNF signaling pathway in hippocampus. The downregulation of hippocampal BDNF expression has been demonstrated previously in various animal depression models and depressed patients, and the chronic treatment of almost all classes of antidepressants increases the expression of BDNF [46]. As an upstream transcriptional activator of BDNF hippocampal CREB expression decreased in experimental animals encountering specific stressors [47, 48]. A decline in CREB expression was also observed in depressed patients [49, 50]. Our results showed that higher doses of GTS treatment (50 and 25 mg kg^−1^ d^−1^) normalized the downregulated hippocampal mRNA and protein levels of BDNF as well as decreasing the activation of CREB in the corticosterone-induced mouse depression model. This result further confirmed the antidepressant-like effects of GTS and suggested that the antidepressant-like effects of GTS may be due to the activation of CREB-BDNF in the hippocampus. Interestingly, our results also indicated that lower doses of GTS (12.5 mg kg^−1^ d^−1^) had no significant antidepressant activity in behavior tests but could improve the expression of BDNF mRNA and phospho-CREB (Ser133) protein in the hippocampus. This result implied that lower doses of GTS had antidepressant potential that was only manifested after prolonged administration.

Previous studies that explored the beneficial effects of ginsenosides on CNS focused on BDNF because of its unique role in CNS. However, the mechanism by which ginsenosides influence the upstream signaling pathway of BDNF has been rarely reported. The efficacy of ginsenosides for preventing age-related memory impairment as well as the increased levels of upstream signaling molecules of the CREB-BDNF pathway including phospho-calcium-calmodulin-dependent kinase II (phospho-CaMKII) and phosphoprotein kinase A Catalytic *β* subunit (phospho-PKA C*β*) in the hippocampus was reported by Zhao et al. [17, 18]. In the present study, we focused on GSK-3*β*, another upstream signaling molecule of CREB-BDNF, because it is involved in various signaling systems [22], and with possible links to mood disorders [21].

Our results on GSK-3*β* and phospho-GSK-3*β* (Ser9) showed that the chronic corticosterone injections increased the GSK-3*β* expression and reduced its inhibitory phosphorylation. This result is consistent with previous studies on CMS-treated rats and depressed patients [28, 29], which further confirms that insufficient GSK-3*β* inhibition is a risk factor for developing depression. In the present study, all three doses of GTS (50, 25, and 12.5 mg kg^−1^ d^−1^) significantly reversed the downregulated inhibitory phosphorylation of GSK-3*β* in this depression model, thereby suggesting that GTS may inhibit GSK-3*β*. To the best of our knowledge, this study is the first to examine hippocampal GSK-3*β* level and activity in this mouse depression model, as well as the first to reveal the effect of GTS on GSK-3*β* in this model. GSK-3*β* is known to participate in several intracellular signaling pathways involving neuroprotection [27]. The results of the present study imply that the GSK-3*β*-CREB signaling pathway may contribute to the decrease of some plasticity-related proteins in the hippocampus and the depression-like behavior. Moreover, the inhibition of the GSK-3*β*-CREB signaling pathway by GTS may account for one of its antidepressant mechanisms. However, our results showed that fluoxetine, which exhibited strong positive effects on the CREB-BDNF signaling pathway, did not significantly alter the GSK-3*β* level or its activity. These findings indicate that GTS and fluoxetine activate the CREB-BDNF signaling pathway using different mechanisms. Previous studies have demonstrated that acute fluoxetine treatment greatly increased the inhibitory serine phosphorylation of GSK-3*β* in the mouse prefrontal cortex [51, 52]. The inconsistencies between the previous findings and our present results suggest that the different brain regions, methods of fluoxetine administration, and animal models influence the effect of fluoxetine on GSK-3*β*.

## 5. Conclusion 

The structural plasticity of the adult hippocampus is critical for the action of antidepressants and the underlying pathophysiology of depression. In the corticosterone-induced mouse depression model, certain doses of GTS exhibit antidepressant-like activities by reversing the decrease of some plasticity-related proteins and activating the CREB-BDNF signaling pathway in hippocampus. The promotion of GSK-3*β* inhibitory phosphorylation which activates the CREB-BDNF signaling pathway may account for the antidepressant-like activity of GTS.

## Figures and Tables

**Figure 1:**
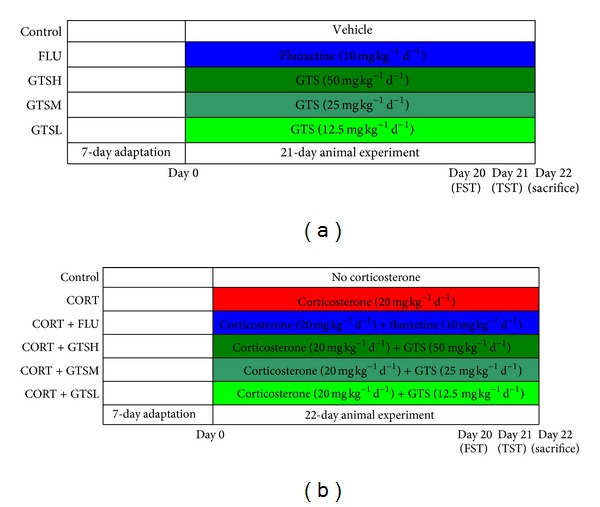
Timeline of the procedure in Experiment 1 (a) and Experiment 2 (b).

**Table 1:** Quality assessment of GTS (*n* = 3).

Extract	Total saponin^a^	Rg1	Re	Rd
	Content (%)	Content (%)	Content (%)	Content (%)
GTS	66.9 ± 1.5	4.9 ± 0.2	3.4 ± 0.1	1.6 ± 0.1

Data are expressed as mean ± SD.

^a^The colorimetric method was used to estimate the GTS content of the samples.
